# The true panel of cystic fibrosis mutations in the Sicilian population

**DOI:** 10.1186/s12881-020-0958-9

**Published:** 2020-05-01

**Authors:** Sandrine Chamayou, Maria Sicali, Debora Lombardo, Elena Maglia, Annalisa Liprino, Clementina Cardea, Michele Fichera, Ermanno Venti, Antonino Guglielmino

**Affiliations:** 1 Unità di Medicina della Riproduzione - Centro HERA, via Barriera del Bosco n. 51/53, 95030 Catania, Sant’Agata Li Battiati Italy; 2 Unit of Gynecology and Obstetric – Department of general surgery and medical surgical specialties, University of Catania, Catania, Italy

**Keywords:** CFTR, Cystic fibrosis, Next generation sequencing, Screening, Sicily

## Abstract

**Background:**

The aim was to establish the true risk of having an affected child with Cystic Fibrosis (CF) in the Sicilian infertile population.

**Methods:**

A longitudinal CFTR screening of 1279 Sicilian infertile patients for all CFTR mutations sequencing the entire gene by Next Generation Sequencing (NGS) was performed from patient’s blood.

**Results:**

One patient out of 16 was a carrier of a CFTR mutation. Twenty-four mutations were found. Theoretically one couple out of 256 was at risk of CF transmission.

**Conclusions:**

The risk of CF transmission is unexpectedly high in Sicily and with a high heterogeneity. Sequencing an entire and long gene such as CFTR makes accessible the true panel of mutations in a specific population and helps better to understand the true risk of having an affected child.

## Capsule

Screening all mutations by sequencing the entire gene of CFTR with NGS makes accessible the real panel of mutations in a specific population and helps better to establish the true risk of having an affected child. In Sicily, 1 infertile patient out of 16 was a carrier of a Cystic Fibrosis mutation and 24 mutations were detected.

## Background

Cystic fibrosis (CF) is the most common autosomal recessive disease in the Caucasian population as one person in 25 is a carrier [1] and the incidence is 1 in 3500 live births [2]. The cystic fibrosis transmembrane conductance regulator (CFTR) gene [3, 4] is responsible for the disease. The gene is located in position 7q31.2, is compound of 27 exons and encodes for the cyclic adenosine monophosphate dependent chloride channel located in the apical membrane of secretory epithelial cells [5, 6]. The transmembrane protein inactivity due to CFTR gene mutation induces hyper-viscosity of epithelial secretions. The first Cystic Fibrosis Mutation Database recorded 2067 genomic variants [7]. On the 11th March of 2019, the number of CF causing mutations has been recalculated as 383; most of the genomic variants have been removed because found in only one patient in the world [8]. The clinical symptomatology of the disease is widely variable from a mild clinical expressivity with atypical pancreatitis and bronchiectasis to severe health consequences including chronic pulmonary obstruction, infections, exocrine pancreatic insufficiency and death [9].

In male patients, the absence or severely reduced activity of CFTR protein can lead to excessive viscosity of the epididymal fluid associated with infertility [10–12] and/or congenital bilateral absence of vas deferens (CBAVD) [13, 14]. Common CFTR mutations such as F508del were found to be involved in the pathogenesis of CBAVD when associated with the variant at the intron 8 poly TG-poly T abbreviated as ‘TG12;5 T’ [15–17].

In 2009, the latest practice European guidelines for molecular genetic diagnosis of CF and CFTR-related disorders were published [18]. At that time, the available methods for mutation detection were based on either the direct gene analysis of known mutations (heteroduplex analysis, restriction enzyme analysis, reverse dot blot hybridization, amplification refractory mutation system, oligonucleotide ligation assay,…) or on the detection of unknown mutations (DGGE, DHPLC, SSCP, gene sequencing, quantitative fluorescent multiplex PCR, MLPA). In Assisted Reproductive Techniques, the approach of CFTR screening is to test the patient(s) for a limited number of known mutations with commercial kits. Nevertheless, nearly the 25% of CF mutations could remain untested and so the resulted percentage of being a carrier would be undervalued [19]. Moreover, the percentage of undetected mutations increases from Northern to Southern Europe [18]. In Italy, twelve mutations are responsible for the main CF genotypes with a frequency from 63 to 85% according to the Region [20].

Recent technological advances such as Next Generation Sequencing (NGS) enlarged the spectrum of detectable mutations [21, 22]. We present the first report of longitudinal screening for CF on Sicilian infertile population by sequencing the entire CFTR gene by NGS.

## Methods

### Patients undergoing CF screening

From July 2014 to June 2019, 1155 couples living in Sicily and coming for infertility counselling were screened for the risk of CF transmission. The entire gene was sequenced by NGS from blood sample of one member of the couple. If the first member resulted as a carrier of CFTR mutation(s) or carrier of the genomic variant TG12;5 T, the CFTR mutations screening was extended to the second member. In total, 1279 Caucasian patients (1055 males and 224 females) were screened. If the couple resulted to be at risk for CF transmission, the principles and protocols of prenatal diagnosis and preimplantation genetic testing (PGT) for CF were explained.

### CF screening from blood

The present CF screening protocol was previously validated by an international network for CFTR gene mutations detection using NGS [21, 23]. It was established that the test had 99.7% of accuracy and 93.8% of specificity. The protocol of CF screening from blood has been previously published [23] and is summarized here: genomic DNA was extracted from peripheral blood using the standard protocol of the High Pure PCR Template Preparation Kit (Roche Diagnostics). 5 ng of genomic DNA from each sample was used to prepare amplicon libraries according to the Ion Ampliseq CFTR Panel (Life Technologies-Thermo Fisher, Carlsbad, USA). All libraries were barcoded, mixed and clonally amplified in OneTouch 2 System. DNA sequencing was performed on the Ion Personal Genome Machine.

The variant analysis was performed using the workflow “*AmpliSeq CFTR single sample”* in Ion Reporter Software. The variant pathogenicity was referred according to referenced database: www.genet.sickkids.on.ce [7]; cftr2.org/mutations_history_CFTR2_11March2019(1).xlsx [8]; varsom.com [24]; www.cftr.iurc.mont.inserm.fr [25]; www.ncbi.nlm.nih.gov/clinvar/ [26, 27].

## Results

### CF screening

Of the 1279 screened patients for CFTR, 71 (5.6%) were diagnosed as a carrier of one CFTR mutation, 1 patient that was asymptomatic for CF was homozygous for D1270N, 1 patient was heterozygote compound (G542X;F1052V, asymptomatic patient), 1 patient was a carrier of F508del/N-TG12;5 T, 1 patient was a carrier of F1052V/N-TG12;5 T and 5 patients were carriers of pathogenic complex alleles. In total, 80 patients were diagnosed as carriers of at least one mutated CFTR gene (6.3% or 1/16), 60 males (5.7% or 1/17.6) and 20 females (8.9% or 1/11.2). Ten infertile couples discovered to be at risk of CF transmission during this screening.

Forty-four patients were carriers of the genomic variant TG12;5 T without CFTR mutation and 7 of benign complex allele. Fifty-four patients carried variants of uncertain significance (VUS): 24 undefined VUS, 4 VUS1, 2 VUS2, 8 VUS3 and 6 VUS4.

The theoretical risk of having an affected child with CF was calculated as 1 couple out of 256 of our Sicilian infertile couples (1/256 = 1/16 × 1/16).

Details of CFTR genotypes are reported in Table 1. 
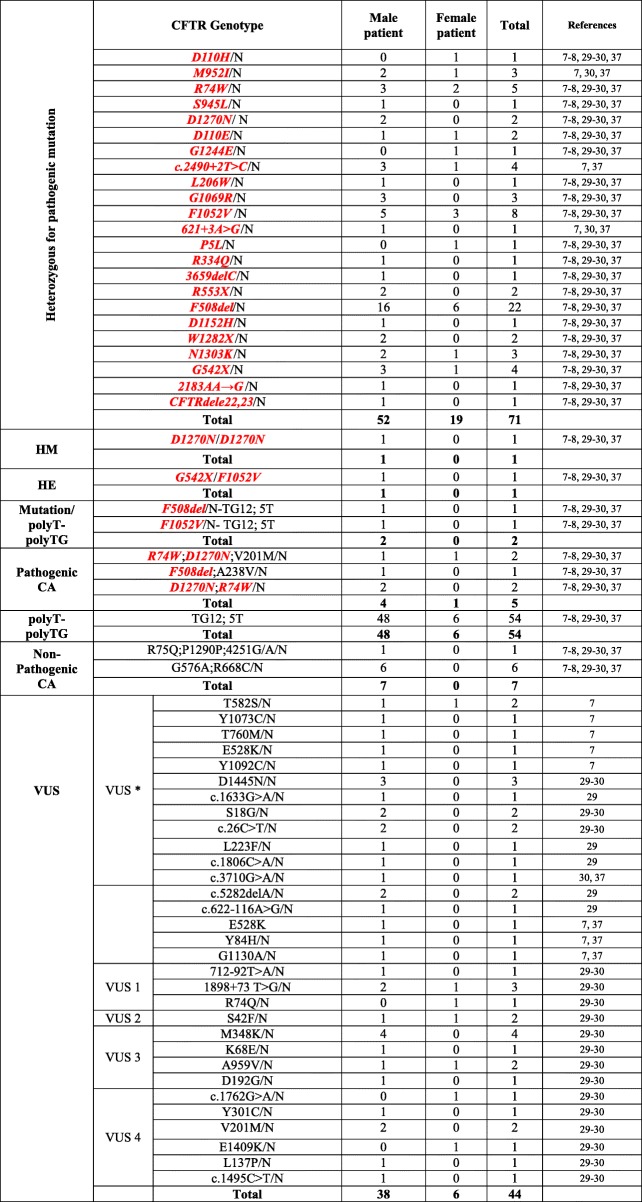

**Table 1:** CFTR Genotype of the tested eastern Sicilian infertile population

	CFTR Genotype		Male patient	Female patient	Total	References
**Heterozygous for pathogenic mutation**	***D110H*/N**		0	1	1	[7, 8, 24–26]
	***M952I*/N**		2	1	3	[7, 25]
	***R74W*/N**		3	2	5	[7, 8, 24–26]
	***S945L*/N**		1	0	1	[7, 8, 24–26]
	***D1270N*/ N**		2	0	2	[7, 8, 24–26]
	***D110E*/N**		1	1	2	[7, 8, 24–26]
	***G1244E*/N**		0	1	1	[7, 8, 24–26]
	***c.2490+2T>C*/N**		3	1	4	[7, 26]
	***L206W*/N**		1	0	1	[7, 8, 24–26]
	***G1069R*/N**		3	0	3	[7, 8, 24–26]
	***F1052V*/N**		5	3	8	[7, 8, 24–26]
	***621+3A>G*/N**		1	0	1	[7, 25, 26]
	***P5L*/N**		0	1	1	[7, 8, 24–26]
	***R334Q*/N**		1	0	1	[7, 8, 24–26]
	***3659delC*/N**		1	0	1	[7, 8, 24–26]
	***R553X*/N**		2	0	2	[7, 8, 24–26]
	***F508del*/N**		16	6	22	[7, 8, 24–26]
	***D1152H*/N**		1	0	1	[7, 8, 24–26]
	***W1282X*/N**		2	0	2	[7, 8, 24–26]
	***N1303K*/N**		2	1	3	[7, 8, 24–26]
	***G542X*/N**		3	1	4	[7, 8, 24–26]
	***2183AA→G*/N**		1	0	1	[7, 8, 24–26]
	***CFTRdele22,23*/N**		1	0	1	[7, 8, 24–26]
	**Total**		**52**	**19**	**71**	
**HM**	***D1270N/D1270N***		1	0	1	[7, 8, 24–26]
	**Total**		**1**	**0**	**1**	
**HE**	***G542X/F1052V***		1	0	1	[7, 8, 24–26]
	**Total**		**1**	**0**	**1**	
**Mutation/ polyT-polyTG**	***F508del*/N-TG12; 5T**		1	0	1	[7, 8, 24–26]
	***F1052V*/N- TG12; 5T**		1	0	1	[7, 8, 24–26]
	**Total**		**2**	**0**	**2**	
**Pathogenic CA**	***R74W; D1270N*;*V201M*/N**		1	1	2	[7, 8, 24–26]
	***F508del*; A238V/N**		1	0	1	[7, 8, 24–26]
	***D1270N; R74W*/N**		2	0	2	[7, 8, 24–26]
	**Total**		**4**	**1**	**5**	
**polyT-polyTG**	TG12; 5T		48	6	54	[7, 8, 24–26]
	**Total**		**48**	**6**	**54**	
**Non-Pathogenic CA**	R75Q;P1290P;4251G/A/N		1	0	1	[7, 8, 24–26]
	G576A;R668C/N		6	0	6	[7, 8, 24–26]
	**Total**		**7**	**0**	**7**	
**VUS**	VUS^a^	T582S/N	1	1	2	[7]
		Y1073C/N	1	0	1	[7]
		T760M/N	1	0	1	[7]
		E528K/N	1	0	1	[7]
		Y1092C/N	1	0	1	[7]
		D1445N/N	3	0	3	[24, 25]
		c.1633G>A/N	1	0	1	[24]
		S18G/N	2	0	2	[24, 25]
		c.26C>T/N	2	0	2	[24, 25]
		L223F/N	1	0	1	[24]
		c.1806C>A/N	1	0	1	[24]
		c.3710G>A/N	1	0	1	[25, 26]
		c.5282delA/N	2	0	2	[24]
		c.622-116A>G/N	1	0	1	[24]
		E528K	1	0	1	[7, 26]
		Y84H/N	1	0	1	[7, 26]
		G1130A/N	1	0	1	[7, 26]
	VUS 1	712-92T>A/N	1	0	1	[24, 25]
		1898+73 T>G/N	2	1	3	[24, 25]
		R74Q/N	0	1	1	[24, 25]
	VUS 2	S42F/N	1	1	2	[24, 25]
	VUS 3	M348K/N	4	0	4	[24, 25]
		K68E/N	1	0	1	[24, 25]
		A959V/N	1	1	2	[24, 25]
		D192G/N	1	0	1	[24, 25]
	VUS 4	c.1762G>A/N	0	1	1	[24, 25]
		Y301C/N	1	0	1	[24, 25]
		V201M/N	2	0	2	[24, 25]
		E1409K/N	0	1	1	[24, 25]
		L137P/N	1	0	1	[24, 25]
		c.1495C>T/N	1	0	1	[24, 25]
		**Total**	**38**	**6**	**44**	

CA: complex allele; HE: heterozygous compound; HM: homozygous; N: wild-type allele; VUS: variant of uncertain significance

*: classification as VUS 1 to 4 has not been determined yet according to The Human Genomics Community (24) and CFTR-France Database (25). Red ink: pathogenic CFTR mutation

*Nota bene*: the variant pathogenicity was referred according to referenced database: www.genet.sickkids.on.ce [7]; cftr2.org/mutations_historyCFTR2_11March2019(1).xlsx [8]; varsom.com [24]; www.cftr.iurc.mont.inserm.fr [25]; www.ncbi.nlm.nih.gov/clinvar/ [26]

## Discussion

Of the 1279 infertile patients tested in our laboratory for the screening of all CF mutations through NGS, 80 (6.3%) were at risk of the transmission of a mutated CFTR gene. One infertile Sicilian patient out of 16 was diagnosed as a carrier of one mutated CFTR gene and it was calculated that 1 couple in 256 was at risk of CF transmission. The carrier frequency (1/16) is 0.6 times more than expected for the Caucasian population which is estimated at 1 on 25 [1].

Twenty-four CFTR mutations were observed. In Sicily F508del was observed in 30.0% (24/80) of CFTR mutations while it can reach up to 44.8% of CF causes in the rest of Italy [28]. The high genetic heterogeneity on the island of Sicily was previously demonstrated for HBB gene [29]. The high allele heterogeneity is explained by the multiple invasions during the centuries especially from Northern populations.

Different commercial kits for first level analysis [30] and NGS-assay screen for a large panel of mutations [31] but none of them test overall CFTR mutations found here. Respectively 12 and 6 of the mutations found in the present work would not have been detected with the strategies described elsewhere [30, 31].

When a commercial test is applied, it is usually performed on both members of the couple in order to minimize the residual risk of being a couple at risk of CF transmission. In Italy, the Society of Human Genetics recommends performing the first level CFTR screening only if one partner was identified as a carrier [32]. In our present strategy the entire CFTR gene is sequenced in only one member of all couples and extended to the second member only if the first member is diagnosed as a carrier. Consequently, the residual risk of being a carrier is drastically decreased and depends only on the discovery of unknown CFTR mutations.

In scientific literature, data are still missing on the clinical signification of variants called VUS [24]. In these cases, the genetic counseling for reproductive choice and genetic selection with prenatal diagnostic or PGT becomes difficult. Genomic variants such as F508C were classified as mutations causing at first [25], then have been subsequently downgraded as benign [8]. Other genomic variants such as T582S are only reported as a mutation on the Cystic Fibrosis Mutation Database and as suspicious VUS according to The Human Genomics Community [24].

CF remains among the widespread diseases tested especially at preimplantation stage [33–35]. In 2016, an international consensus was found for the best practice for PGT of CF [36]. We developed and clinically applied a universal strategy for PGT based on NGS to diagnose CF mutations found in Sicily [23].

## Conclusion

CF is the most widespread autosomal recessive disease present in the Caucasian population with a very large allele variability as 383 mutations have been recently listed and many genomic variations have clinical consequences to ascertain. In the present work, we showed how the longitudinal screening of the entire CFTR gene is determinant for the detection of all the mutations within a specific population. In our tested Sicilian infertile population, the theorical risk of being a carrier was 1/16, that is 0.6 times more than the 1/25 Caucasian reference. Consequently, it was calculated that one couple out of 256 was at risk of having an affected child. At least 24 CFTR gene mutations are present in the Sicilian population.

## Data Availability

The data of the manuscript are available in: https://apps.thermofisher.com/apps/spa/#/dashboard; Username: hera.sharedata@gmail.com; password: sharedata2020.

## Abbreviations

CBAVD: Congenital Bilateral Absence of Vas Deferens
CF: Cystic fibrosis
CFTR: Cystic Fibrosis Transmembrane Regulator
IGV: Interactive Genomic Viewer
NGS: Next-Generation Sequencing
PGT: Preimplantation Genetic Testing
VUS: Variances of Uncertain Significance
